# pH salivary analysis of subjects suffering from Sjögren's Syndrome and laryngopharyngeal reflux

**DOI:** 10.1590/S1808-86942012000100013

**Published:** 2015-10-20

**Authors:** Marco Antonio dos Anjos Corvo, Claudia Alessandra Eckley, Bianca Maria Liquidato, Gustavo Leão Castilho, Cibelle Nunes de Arruda

**Affiliations:** a MSc in Medicine – Medical Sciences School - Santa Casa de São Paulo (Second Assistant Professor – Department of Otorhinolaryngology of the Santa Casa de Misericórdia hospital of São Paulo); b PhD in Medicine – Medical Sciences School - Santa Casa de São Paulo (Assistant Professor – Department of Otorhinolaryngology of the Santa Casa de Misericórdia hospital of d São Paulo); c PhD in Medicine – Medical Sciences School - Santa Casa de São Paulo (Assistant Professor – Department of Anatomy and Physiology of the Santa Casa de Misericórdia hospital of São Paulo); d 2^nd^ Year Resident in Otorhinolaryngology - Santa Casa de Misericórdia de São Paulo.; e 1^st^ Year Resident in Otorhinolaryngology - Santa Casa de Misericórdia de São Paulo. Departamento de Otorrinolaringologia da Faculdade de Ciências Médicas da Santa Casa de São Paulo.

**Keywords:** gastroesophageal reflux, saliva, laryngeal diseases, xerostomia

## Abstract

Saliva is one of the components for the digestive homeostasis. Recent studies have shown that patients with laryngopharyngeal reflux (LPR) present a drop in salivary pH. Patients with Sjögren´s syndrome (SS) are a potential clinical research model for xerostomia and its laryngeal and pharyngeal consequences. The aim was to evaluate the characteristics of saliva of patients with SS and LPR.

**Methods:**

19 patients with SS plus LPR, and 12 healthy controls had their saliva studied prospectively for volume and pH. Two salivary samples were obtained from each participant: whole unstimulated saliva(WUS) and whole stimulated saliva(WSS) while chewing parafilm M^®^. All the participants were females.

**Results:**

Mean age was 60 years (study group) and 44 years (control). LPR was diagnosed on all 19 subjects. The mean pH of WUS was 7.53 (SS) and 7.57 (controls), raising to 7.87 and 7.83 respectively after stimulation. The mean salivary volume of patients with SS was 1.27 mL (WUS) and 3.78 mL (WSS), whereas controls had a significantly higher salivary volume both before and after stimuli.

**Conclusion:**

A very high prevalence of LPR was found in patients with SS, which is probably caused by a uniform drop in salivary volume and all its contents, rather than a specific deficiency in its components, as shown previously in patients without SS.

## INTRODUCTION

Saliva plays a fundamental role in the digestive system's homeostasis, for its inorganic content - made up of water and bicarbonate ions, but primarily by its organic and protein make up^1^. Thus, one might question the clinical situation caused by xerostomia on the basic salivary functions of food bolus lubrication, on the beginning of the enzymatic digestion, buffering capacity and local immune response^1^. Previous studies indicate that low saliva volumes reduce the esophageal-salivary reflex effectiveness and could contribute to the occurrence of oral, esophageal and dyspeptic diseases^2^, ^3^.

Xerostomia is defined as a subjective sensation of dry mouth (from the Greek xeros - dry stoma - mouth)^4^, ^5^. It may be present in various diseases, among which we stress Sjögren's syndrome (SS)^1^, ^4^, ^5^, ^6^, ^7^, ^8^, ^9^, ^10^. Of probable autoimmune origin, SS affects the exocrine glands, which undergo progressive lymphoplasmacytic infiltration and leads to functional failures^9^, ^10^. Since the salivary glands are frequently affected by infiltration, the disease is recognized for causing clinically noteworthy xerostomia^9^, ^10^.

Some authors have reported higher prevalence of gastroesophageal reflux disease (GERD) in populations with SS^11^, ^12^, ^13^. However, with regards to laryngopharyngeal reflux (LPR), only rare reports mention its occurrence as frequent in subjects with SS, with no specific studies designed to establish this correlation^14^, ^15^, ^16^, ^17^.

For individuals with xerostomia, Eckley & Costa^18^ noticed significant drops in the pH and in the salivary volume of patients with LPR, when compared to normal individuals. Moreover, these changes seem to be a response to reflux, and not a primary deficiency, because after controlling the disease, pH and salivary volume go back to normal^19^. However, in SS individuals, it is not known how saliva could interfere in the balance of the laryngeal and esophageal coating epithelium. Would there be local tissue damage simply because of the volumetric reduction in salivary volume or would there be changes in the salivary make up involved in the genesis and maintenance of the findings?

Our hypothesis is that there would be some specific change in the saliva of individuals with SS which would cause a reduction in laryngeal and pharyngeal protection, making some of these individuals more susceptible to LPR, as it seems to happen with individuals without xerostomia^18^, ^20^. The early identification of these individuals in risk by studying their salivary characteristics could be of high importance for preventing the complications associated with the laryngopharyngeal reflux, enhancing the quality of life of individuals with Sjögren's syndrome.

The present paper aims at comparing the salivary volume and pH of individuals with Sjögren's syndrome and laryngopharyngeal reflux to those of healthy individuals.

## MATERIALS AND METHODS

After approval by the Ethics in Research with Human Beings' Committee of our institution (Project number 034/07), a total of 36 adult patients diagnosed with Sjögren were investigated in a cross-section manner. The patients were consecutively and randomly selected by telephone call, from the database of the stomatology wards of a tertiary university hospital, between January 2007 and December of 2009. We admitted all the patients who met the inclusion and exclusion criteria, after a statement about the objectives, methodologies and risks.

Inclusion factors were: adult patients with hyposalivation and a confirmed diagnosis of Sjögren's syndrome by the current diagnoses criteria of the American-European consensus^21^.

The exclusion factors were other conditions which could cause chronic laryngopharyngitis, such as: smoking, drinking alcoholic beverages, exposure to abrasive inhaling chemicals and overt allergic or infectious agents^20^. In addition, we took off the study those patients who were unable to produce a minimum amount of saliva for sampling and biochemical analysis, patients whom we could suspend prokinetic or proton pump inhibitor drugs, patients submitted to salivary gland surgery and patients with pre-neoplastic and neoplastic laryngeal and pharyngeal lesions (current or previously treated)^18^, ^20^, ^22^.

Of the initially 36 selected patients with SS by the criteria used, 17 were withdrawn from the study by the conditions described in Table 1.

**Table 1:** Reasons for excluding patients with Sjögren's syndrome initially selected by the study.

Abandoning the protocol	5
Insufficient saliva production for analysis purposes	3
C Hepatitis diagnosed during the protocol	3
Neoplasia diagnosed during the protocol	2
Return to smoking during the protocol	2
Death before finishing the protocol	1
Previous gastric fundoplication	1

**Total**	17

The patients answered a detailed questionnaire about laryngopharyngeal and digestive symptoms suggestive of GERD and LPR, and subsequently underwent a thorough ENT examination and rigid laryngoscopy (without topical anesthesia). We used the flexible nasallaryngoscope only in cases of hyperreflexia or impossibility to visualize the segment with the telescope.

Two consecutive samples of whole saliva were obtained from each participant: the first, unstimulated whole saliva (UWS) and second, stimulated whole saliva (SWS), mechanically by chewing a 25 cm^2^ piece parafilm M^®^ (Pechiney Plastic Packing Chicago IL, USA)^23^.

Collection was always performed in the morning and with the patient fasting for at least eight hours. The patient was instructed not to brush his/her teeth or use mouthwash in the morning of saliva collection. The method of collection was the one previously described by Eckley et al.^18^, ^19^, ^20^

The UWS and SWS samples were collected by asking the patient to “spit” freely in a collection bottle the entire saliva volume produced during the 10-minute period. To preserve their rheological and biochemical characteristics, salivary samples were stored in a refrigerator at 5 degrees Celsius, until they could be processed and analyzed.

The processing was done in the same saliva collection period by centrifugation for 10 minutes (3500 rpm – Excelsa II centrifuge - Fanem - Brazil) for sedimentation of cellular debris; the supernatant was separated for pH and volume measurement. Salivary volume measurement was made using pipettes and graduated test tubes, being recorded in milliliters^24^. Salivary pH was measured using a digital pH meter (Denver Instrument Company, Model: Basic pH meter, Arvada, CO, USA).

The LPR diagnosis was made based on laryngeal signs and symptoms, using two research instruments, the rate of reflux symptoms (reflux symptom index - RSI)^25^, and the Reflux Finding Score (RFS)^26^, both previously validated in the literature for the English language^25^, ^26^. Symptoms which added scores were equal to or higher than 13 in the RSI^25^ were considered positive for LPR, and laryngeal signs which added scores were higher than or equal to seven in the RFS^26^.

Laryngopharyngeal symptoms and direct laryngoscopy signs matched the results of endoscopy and/or dual-channel 24-hour esophageal pH monitoring.

With technical support from a specific professional, the results were plotted and analyzed using parametric and non-parametric statistical methods for variable analyses (Student's t test and Wilcoxon test). We considered a “*p*” value lower than or equal to 0.05 as significant.

To determine the importance of the results for the SS group examined we also studied a group of 12 healthy female volunteers, who were selected by following the same exclusion criteria set forth above. Moreover, none of the volunteers met the diagnostic criteria used for SS. All were also submitted to RSI^25^ and RFS^26^ and to rule out the presence of LPR, a condition for participating in the control group. Saliva collection and the salivary parameters studied were similar to those described for the study group.

## RESULTS

All the 19 individuals in this study were females, with a mean age of 60 years (varying between 49 and 74 years). Nine patients had primary SS, seven cases were secondary to rheumatoid arthritis, two were secondary to systemic lupus erythematous and one to scleroderma. The mean diagnosis time for the individuals with SS at the time when the present study was analyzed was 58 months (4 years and 10 months), with a median of 5 years and 4 months and standard deviation of 2 years and 5 months.

The control group was made up of 12 healthy women, with mean age of 44 years (26 to 62 years), median of 42 years and standard deviation of 11.5; without laryngeal signs or symptoms of LPR. Mean RSI was zero and the mean RFS was 1.75, varying between zero and three points, both confirming that there was no posterior laryngitis.

There was a difference between the mean age of the study group and that of the control group. Even then, when we correlated the age of all the individuals (study and control groups) with the quantitative salivary variables studied (volume and pH), the Pearson's correlation index showed low values, indicating that both groups could be compared vis-à-vis these variables (Table 2).

**Table 2:** Pearson's correlation index values in the comparison between the age of the individuals and the salivary samples investigated, in the total non-stimulated saliva (TNSS) and total stimulated saliva (TSS).

	TNSS		TSS	
Compared variable	Volume	pH	Volume	pH

Pearson's correlation	-0.370	-0.047	-0.419	0.054

In the study group, the mean RSI was 19.5, varying between 13 and 30 points. The mean RFS was 11.56, varying between eight and eighteen points.

Of the eight patients with reflux esophagitis in the study group, four had non-erosive esophagitis and four and the erosive type (Los Angeles grade A), there was no one with Barret's esophagus in the series. Because they had RSI and RFS suggestive of laryngopharyngeal reflux, with UGE (upper gastrointestinal endoscopy) confirming gastroesophageal disease, in eight individuals we confirmed the laryngopharyngeal reflux. In the remaining 11 individuals in whom UGE was not changed, we did manometry and esophageal pH measuring, according to the methodology utilized.

As to the esophageal manometry, seven patients had a normal exam, two patients had hypotonia of the lower esophageal sphincter, and two had mild esophageal dysmotility. However, as to the double-sensor esophageal pH probe study, the result was uniform, because all the 11 patients in the study group had pathological reflux episodes in the proximal sensor, making up the LPR diagnosis. Only three of these individuals had reflux in pathological levels also in the distal sensor, which configures GERD and LPR together.

When we analyzed the individuals with SS as to disease subtype (primary or secondary), we did not notice a statistically significant difference, by the Mann-Whitney test, in characterizing the sample as to age (*p*=0.549), as to RSI (*p*=0.447) and as to RFS (*p*=0.400). Since the SS subtypes were statistically equal for the salivary variables analyzed (volume and pH), we considered the individuals with SS in the same group in order to compare them with the control group.

**Figure 1:**
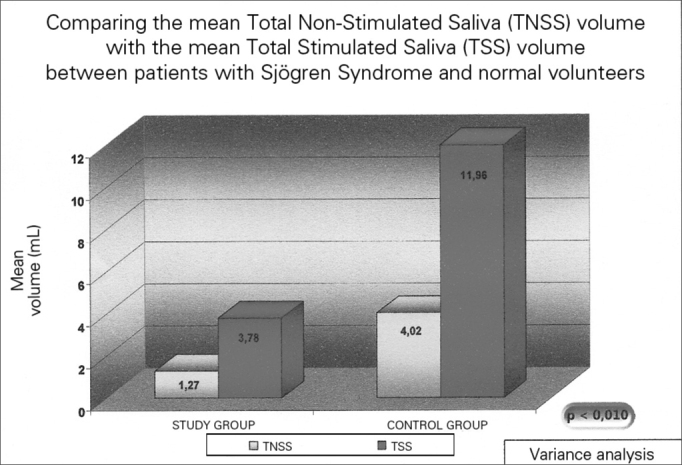
Salivary mean volume in individuals with Sjögren (study group) and in healthy individuals - TNSS- Total Non-Stimulated Saliva TSS – Total Stimulated Saliva.

**Figure 2:**
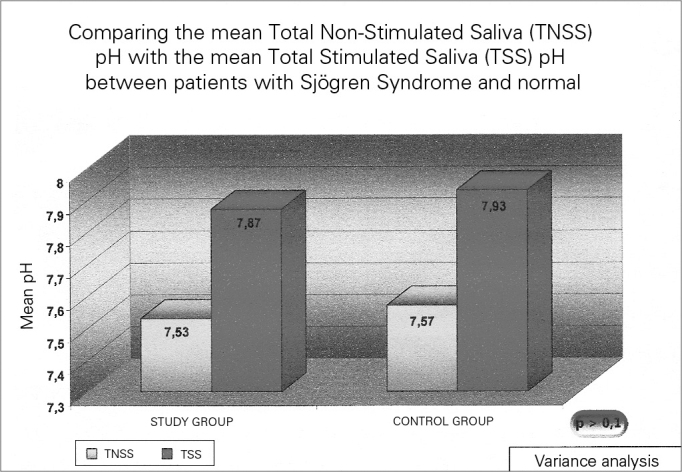
Mean salivary ph in individuals with Sjögren (study group) and in healthy individuals – TNSS – Total Non-Stimulated Saliva TSS- Total Stimulated Saliva.

Salivary pH and volume results are presented on Figures 1 and 2, and are described below.

The total unstimulated saliva volume (TUSV) of the individuals with SS varied between 0.1 and 4.1 mL (mean value of 1.27 mL) (sd=1.06). The mean Total Stimulated Saliva (TSS) volume was 3.78 mL, varying between 0.1 and 10.3 mL (sd=2.87). This difference was statistically significant (*p*=0.009). TUSV pH in patients with SS showed a mean value of 7.53, varying between 6.40 – 8.40 (sd=0.51), and TSS was 7.87, varying between 6.67 and 8.43 (sd=0.47), and this difference was also statistically significant (*p* =0.002).

In the group of healthy volunteers, the mean salivary volume of the NSSV was 4.02, varying between 1.6 and 8.0mL, and the STS was 11.96 mL, varying between 4.5- 19.0mL (*p*=0.002). Now, the mean NSTS pH was 7.57, varying between 6.83 and 7.92; and the mean value of the TSS was 7.93, varying between 7.56 and 8.17 (*p*=0.004). All the above-studied variables in the control group showed statistically significant differences between the stimulated and the non-stimulated salivary samples.

As expected, SS patients had a statistically lower NSTS and STS mean volumes when compared to the control individuals (*p*<0.001). Nonetheless, there was no statistically significant difference in the saliva pH between the study and the control groups, both for the total non-stimulated and the stimulated total saliva (*p*>0.1).

## DISCUSSION

The motivation to go more in-depth in understanding this topic stemmed from the need to understand LPR genesis more in-depth, and in the empirical observation of some patients with LPR being followed up, who had hyposalivation, according to the studies carried out by Eckley, in 2004^19^. Thus, the project was conceived to conjugate the research objectives in salivary biochemical factors in the LPR genesis, with the need to offer our SS patients a better understanding of their disease and all the manifestations surrounding them.

For the LPR diagnosis, we considered the laryngoscopic signs and symptoms translated by the RSI^25^ and RFS^26^ instruments, respectively; nonetheless in association with the UDTE, esophageal pressure and 24-hour double channel esophageal pH probe data. We agree with the current trend that the RFS seems to be a good index to assess the presence of endoscopic signs of laryngitis, without this laryngeal inflammation being necessarily linked to reflux of the gastroduodenal content such as etiology (reason to adopt such strict exclusion criteria)^17^, ^22^. Thus, the present study agrees with the recent publications by Gupta & Sataloff^17^ and Ali^22^ as admitting to a suspicion of LPR only when the RFS values higher than seven were associated with characteristic symptoms, with the RSI greater than 13 which still relates to the patient's clinical complaint as an important suspicion for diagnosis. Even if ideal, the conjugation of these two instruments was paramount to establish the LPR suspicion, which was later corroborated by UDTE and the double channel 24 hour esophageal pH probe.

Although rare, the literature had already considered them possible laryngeal manifestations of the SS. In a 2003 publication, Belafsky & Postma^11^ empirically recognized a high prevalence of LPR and esophageal dysmotility in patients with SS^11^. In the current series, only two of the 19 patients with SS had esophageal dysmotility.

In 2005, Ogut et al.^12^, submitted 77 patients with SS to RSI and RFS search criteria, concluding that in this subgroup, the score values were significantly higher when compared to the normal individuals studied. The present study achieved great notability because of the expressive series reported, and it also stimulated the current study to try to understand the reason for such a high referred association.

Therefore, considering the previous literature data, and considering the protective properties of saliva to the digestive system^18^, ^20^, we expected to find laryngopharyngeal changes in patients with SS. The initial hypothesis was that the association with SS with LPR would happen because of qualitative and not quantitative salivary deficiency. However, the current series did not allow to establish a correlation, since all the patients studied had LPR.

Analyzing the salivary data, we noticed that the mean volume of the TNSS samples from patients with SS was really low (1.27mL), with pH values slightly alkaline (mean value of 7.53). Even if these patients suffer from a saliva-production-limiting disease, the masticatory stimulation employed was able to increase the saliva volume (mean of 3.78mL), with a statistically significant difference. The mean TSS pH value went up to 7.87, reflecting a greater alkalinity of the fluid after the stimulus. This pH increase is particularly relevant for SS patients, if we consider the large prevalence of LPR seen in the study and the need for these patients to, theoretically, having greater efficiency in salivary buffering.

Since all SS patients had a behavior similar to that of LPR, comparing TNS and TSS variables of individuals with SS and without LPR were fundamental in the attempt to interpret the findings.

In relation to the results from the volumes obtained, the TNSS volume from SS individuals was reduced, showing a mean value of 1.27mL in a 10-minute collection. This value was statistically lower when compared to that from the control group, as to be expected for populations with SS.

About the pH of the salivary samples, it was noticed that the masticatory stimulus did not cause a statistically significant difference within the groups analyzed (control and study). Moreover, when compared to healthy individuals, the salivary samples of individuals with SS who had statistically equal pH values. Thus, differently than patients with LPR and without xerostomia, who had more acid saliva pH values in comparison with normal individuals, the patient with SS and with LPR did not have this altered mean pH value^19^, ^20^, ^27^. In other words, changes in salivary pH did not seem to be involved in the greater occurrence of LPR in patients with SS, suggesting a different pathophysiology from the LPR for this special group.

The rarity of SS in the world and the lack of official data both on disease prevalence as to its other particularities in Brazil make the recording of the reflux behavior in this subpopulation a topic very little explored before^9^, ^10^. The results presented in this study represent part of an ongoing study about the organic and inorganic characteristics of patients with SS, and its influence in the genesis of LPR and GERD. Thus, greater details on the pathophysiology of the disease in this subgroup of patients with SS are awaited, as new data become available.

## CONCLUSIONS

Based on the present study, we concluded that there was no statistically significant difference in the salivary pH of individuals with Sjögren syndrome and healthy individuals, both in the non-stimulated total saliva as well as in the total stimulated saliva. This suggests that the laryngopharyngeal reflux may have a unique pathophysiology in SS individuals, possibly associated with the global drop in salivary volume and all its components.
